# Supplementary material for: Non-random host tree infestation by the Neotropical liana Marcgravia longifolia
Source: PeerJ. 2022 Dec 15;10:e14535. doi: 10.7717/peerj.14535 (PMC9760024; doi:10.7717/peerj.14535)
Supplement: Supplemental Information 2 — CI: credible interval [file peerj-10-14535-s002.pdf]

Supplementary Table 2.

Estimated species specific infestation probabilities (inverse logit transformed estimated Best Linear Unbiased Predictors plus the intercept). CI: credible interval

**Tree genus**	**lowerCI99**	**lowerCI50**	**median**	**upperCI50**	**upperCI99**
Abarema	0.000001	0.000111	0.000310	0.001189	0.018174
Albizia	0.000001	0.000110	0.000314	0.001226	0.019040
Anaueria	0.000001	0.000120	0.000318	0.001360	0.021173
Aniba	0.000001	0.000116	0.000310	0.001256	0.020191
Anthodiscus	0.000001	0.000104	0.000305	0.001167	0.019709
Aptandra	0.000001	0.000119	0.000275	0.001178	0.011150
Brosimum	0.003162	0.009233	0.011908	0.016785	0.032892
Buchenavia	0.000036	0.000675	0.001146	0.002732	0.010005
Byrsonima	0.000001	0.000113	0.000315	0.001253	0.018161
Capparis	0.000001	0.000101	0.000248	0.000914	0.008483
Cariniana	0.000053	0.001072	0.001888	0.004960	0.020238
Chrysochlamys	0.000001	0.000101	0.000291	0.001089	0.014662
Chrysophyllum	0.000588	0.006248	0.010359	0.021750	0.071309
Composneura	0.000001	0.000124	0.000312	0.001325	0.018514
Conceveiba	0.000041	0.000812	0.001409	0.003613	0.014693
Couepia	0.000001	0.000096	0.000237	0.000869	0.007841
Couma	0.000001	0.000113	0.000317	0.001238	0.018254
Crematosperma	0.000001	0.000127	0.000271	0.001282	0.011357
Crepidospermum	0.000001	0.000113	0.000312	0.001233	0.017090
Cybianthus	0.000001	0.000128	0.000298	0.001289	0.014122
Dacryodes	0.000002	0.000103	0.000263	0.000978	0.012406
Dendropanax	0.000001	0.000117	0.000310	0.001285	0.018644
Dialium	0.000067	0.001277	0.002516	0.006415	0.030026
Diclinanona	0.000001	0.000093	0.000228	0.000827	0.006760
Duguetia	0.000001	0.000112	0.000310	0.001217	0.016292
Duroia	0.000001	0.000103	0.000268	0.000934	0.009623
Ecclinusa	0.000001	0.000106	0.000248	0.000970	0.008504
Eschweilera	0.003677	0.005149	0.005698	0.006429	0.008620
Ferdinandusa	0.000001	0.000120	0.000289	0.001206	0.013572
Guarea	0.000001	0.000074	0.000149	0.000537	0.003408
Guatteria	0.000001	0.000108	0.000261	0.001060	0.009420
Heisteria	0.000001	0.000107	0.000247	0.000981	0.008604
Helicostylis	0.000056	0.000932	0.001854	0.004260	0.020230

Hevea	0.001579	0.006379	0.008843	0.013779	0.031855
Huberodendron	0.000001	0.000108	0.000311	0.001226	0.018053
Hymenaea	0.004588	0.013097	0.016646	0.023930	0.047992
Inga	0.000001	0.000087	0.000161	0.000633	0.003932
Iryanthera	0.000020	0.000254	0.000402	0.000878	0.003024
Jacaranda	0.000001	0.000112	0.000234	0.001004	0.007658
Ladenbergia	0.000001	0.000095	0.000194	0.000762	0.005397
Leonia	0.000001	0.000112	0.000254	0.001044	0.009699
Licania	0.000263	0.000908	0.001198	0.001785	0.003952
Licaria	0.000001	0.000091	0.000218	0.000792	0.006714
Mabea	0.000059	0.001088	0.002082	0.005462	0.025961
Macrolobium	0.000001	0.000112	0.000250	0.001050	0.010687
Manilkara	0.000001	0.000114	0.000311	0.001247	0.018189
Maprounea	0.000001	0.000134	0.000312	0.001425	0.015172
Matayba	0.000001	0.000110	0.000272	0.001136	0.012347
Matisia	0.000001	0.000096	0.000230	0.000859	0.007608
Memora	0.000001	0.000121	0.000313	0.001265	0.016934
Mezilaurus	0.000001	0.000111	0.000222	0.001015	0.006712
Miconia	0.000001	0.000111	0.000298	0.001101	0.012923
Micrandra	0.000001	0.000062	0.000115	0.000386	0.001768
Micropholis	0.000001	0.000105	0.000223	0.000898	0.006827
Moronobea	0.000001	0.000110	0.000296	0.001182	0.012756
Mouriri	0.000001	0.000097	0.000266	0.000917	0.009284
Naucleopsis	0.000001	0.000109	0.000258	0.001066	0.010106
Neea	0.000001	0.000107	0.000225	0.000904	0.006999
Ocotea	0.000001	0.000096	0.000187	0.000791	0.004921
Oenocarpus	0.000001	0.000063	0.000118	0.000400	0.002143
Ophiocaryon	0.000001	0.000090	0.000206	0.000737	0.006490
Parinari	0.000001	0.000107	0.000318	0.001148	0.018906
Parkia	0.000403	0.001965	0.002733	0.004571	0.010686
Persea	0.000001	0.000122	0.000311	0.001359	0.017268
Pleurothyrium	0.000001	0.000105	0.000306	0.001136	0.017769
Pouroma	0.000001	0.000079	0.000168	0.000573	0.003974
Pouteria	0.002195	0.005845	0.007105	0.010008	0.018245
Protium	0.000001	0.000061	0.000114	0.000383	0.001984
Pseudolmedia	0.000001	0.000118	0.000314	0.001264	0.020207
Rhigospira	0.000001	0.000108	0.000290	0.001115	0.013910
Rinorea	0.000001	0.000117	0.000293	0.001188	0.013287
Sacoglottis	0.000001	0.000117	0.000285	0.001205	0.014466

Simaba	0.000001	0.000113	0.000259	0.001088	0.010720
Sloanea	0.000240	0.001058	0.001509	0.002399	0.005624
Sterculia	0.000001	0.000098	0.000295	0.000996	0.012498
Swartzia	0.000001	0.000081	0.000193	0.000654	0.005189
Tachigali	0.000001	0.000078	0.000148	0.000562	0.002920
Tapirira	0.000001	0.000105	0.000260	0.001010	0.010663
Tetrastylidium	0.000001	0.000093	0.000213	0.000793	0.006303
Toulicia	0.000001	0.000103	0.000237	0.000950	0.007526
Tovomita	0.000001	0.000117	0.000263	0.001105	0.010317
Trichilia	0.000048	0.000961	0.001654	0.004415	0.016940
Vantanea	0.000001	0.000099	0.000198	0.000792	0.004959
Virola	0.000040	0.000611	0.001082	0.002519	0.009848
Xylopia	0.000001	0.000093	0.000246	0.000848	0.008620
Zygia	0.000001	0.000075	0.000165	0.000563	0.003497
